# Development of β-Ga_2_O_3_ layers growth on sapphire substrates employing modeling of precursors ratio in halide vapor phase epitaxy reactor

**DOI:** 10.1038/s41598-020-79154-9

**Published:** 2020-12-17

**Authors:** Galia Pozina, Chih-Wei Hsu, Natalia Abrikossova, Mikhail A. Kaliteevski, Carl Hemmingsson

**Affiliations:** 1 Department of Physics, Chemistry and Biology (IFM), Linköping University, 581 83 Linköping, Sweden; 2 St-Petersburg Academic University, Khlopina 8/3, St. Petersburg, Russian Federation 194021; 3 ITMO University, Kronverkskiy pr. 49, St. Petersburg, Russian Federation 197101; 4 Ioffe Institute, Politekhnicheskaya 26, St. Petersburg, Russian Federation 194021

**Keywords:** Materials for devices, Design, synthesis and processing, Semiconductors

## Abstract

Gallium oxide is a promising semiconductor with great potential for efficient power electronics due to its ultra-wide band gap and high breakdown electric field. Optimization of halide vapor phase epitaxy growth of heteroepitaxial $\upbeta$-Ga_2_O_3_ layers is demonstrated using a simulation model to predict the distribution of the ratio of gallium to oxygen precursors inside the reactor chamber. The best structural quality is obtained for layers grown at 825–850 °C and with a III/VI precursor ratio of 0.2. Although the structural and optical properties are similar, the surface morphology is more deteriorated for the $\upbeta$-Ga_2_O_3_ layers grown on 5 degree off-axis sapphire substrates compared to on-axis samples even for optimized process parameters. Cathodoluminescence with a peak at 3.3 eV is typical for unintentionally doped *n*-type $\upbeta$-Ga_2_O_3_ and shows the appearance of additional emissions in blue and green region at ~ 3.0, ~ 2.8, ~ 2.6 and ~ 2.4 eV, especially when the growth temperatures is lowered to 800–825 °C. Estimation of the band gap energy to ~ 4.65 eV from absorption indicates a high density of vacancy defects.

## Introduction

Gallium oxide (Ga_2_O_3_) is an ultra-wide band gap semiconductor gaining today much attention due to its high breakdown electric field, which is essential for modern high voltage, energy efficient high power electronics and optoelectronics^1^. Ga_2_O_3_ can be synthesized in several different phases ($\alpha ,\beta ,\varepsilon ,\delta ,\gamma ,\kappa$) and even more polymorphs are predicted from first-principles calculations^2^. Monoclinic $\upbeta$-phase with space group (C2/m) is stable and, so far, most studied from point of view of optical, structural and electronic properties^3–6^. $\upbeta$-Ga_2_O_3_ with the band gap energy of ~ 4.8 eV has the potential for better performance than SiC or GaN, not to mention silicon, for ultra-high voltage switching applications^7^. According to Baliga’s figure of merit (BFOM), the combined properties of $\upbeta$-Ga_2_O_3_ are ~ 3000 times better than for silicon in terms of power devices^8^. The break down fields for $\upbeta$-Ga_2_O_3_ is ~ 8 MV/cm, which is larger than for GaN or SiC, while on-resistance at a given breakdown voltage is lower^8^.

Fabrication of electronic devices demands high quality single crystalline Ga_2_O_3_ layers with thicknesses of several microns^9^. Currently, many efforts are focused on the development of suitable epitaxial layers. Growth of $\upbeta$-Ga_2_O_3_ thin films with quality appropriate for electronic applications has been demonstrated by molecular beam epitaxy (MBE)^8,10^. Metal–organic vapor phase epitaxy (MOVPE) is another technique used for manufacturing of homoepitaxial^11^ and heteroepitaxial Ga_2_O_3_ layers^12,13^. However, the growth rates associated with these methods are typically very low (< 1 μm/h), which limits their suitability for growth of thick layers necessary for high power devices. Halide vapor phase epitaxy (HVPE) is a promising technique to produce thick Ga_2_O_3_ layers^14–17^ due to high growth rates up to 250 µm/h as recently demonstrated by Oshima et al.^17^. Although there are commercial native substrates, for example, from Tamura Corporation, the development of heteroepitaxial growth methods on foreign substrates such as sapphire is still relevant due to the lower cost and availability.

In this work, we report on the development of the HVPE process for the growth of $\upbeta$-Ga_2_O_3_ epitaxial layers on (0001) sapphire substrates and compare results with growth using 5 degree off-axis sapphire substrates. To optimize the process, numerical simulations were performed to evaluate the distribution of the precursor ratio at the sample holder. The obtained layers were characterized by X-ray diffraction (XRD), scanning electron microscopy (SEM), cathodoluminescence (CL) and optical absorption.

## Results and discussion

**Figure 1:**
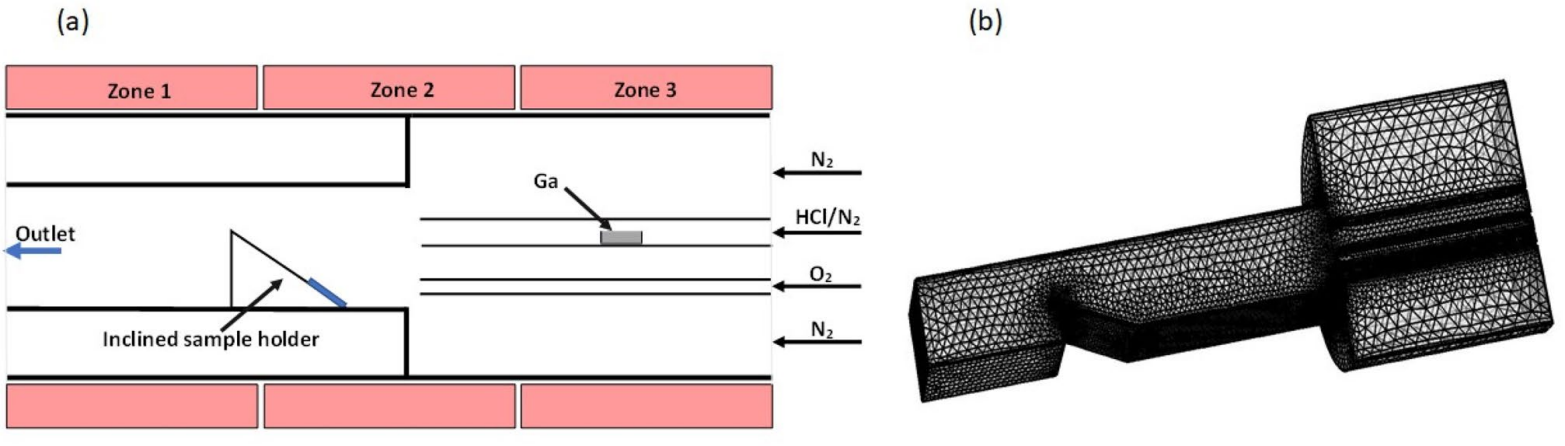
(**a**) Schematic drawing of the HVPE reactor (cut by the vertical plane along the reactor) used as a model in simulations. (**b**) Gradient of mesh used in simulations.

The development of growing $\upbeta$-Ga_2_O_3_ was conducted in a horizontal HVPE reactor of our own design. Previously, we used HVPE growth for producing thick GaN layers in vertical reactor^18,19^, while here we exploit a horizontal chamber consisting of a quartz tube inside a furnace with three temperature zones. Epitaxial growth is affected by many different process parameters such as the geometry of the chamber and gas inlet/outlet, distance to the sample holder, heating temperature and its gradient in the reactor, gas flows, precursors ratio, pressure, etc. We can usually control rather accurately growth temperature, pressure in the reactor, substrate position and total carrier and precursor gas flows. However, the distribution of the precursors ratio within the reactor is more difficult to predict; therefore, numerical calculations of the precursor ratio can be very useful in process optimization. A schematic drawing of the growth zone of the reactor and simulated geometry is shown in Fig. 1a and the gradient mesh in Fig. 1b. The model for simulations is described in Methods section. We used the same inlet gas temperature of 850 °C when calculating the precursor distribution for different gas flow ratios. The reactor was considered as three-dimensional (3D) with a vertical plane of symmetry; thus, to speed up the calculations only half of the reactor was used for simulation.

**Figure 2:**
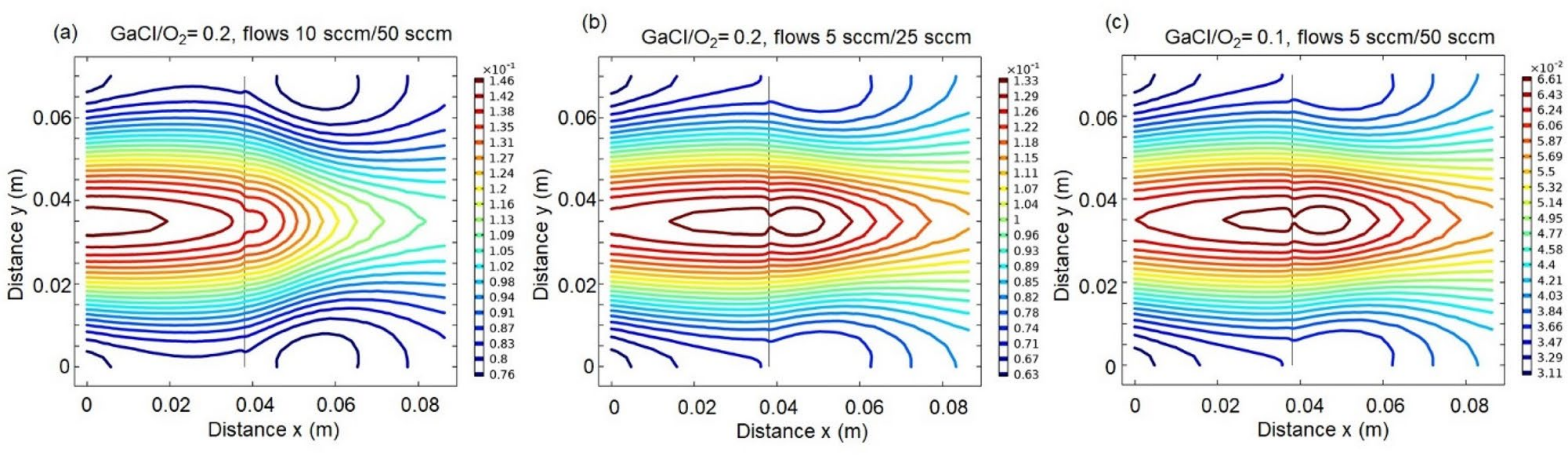
2D distribution of the ratio of GaCl to oxygen within the sample holder for different GaCl and oxygen inlet gas flows of (**a**) 10 sccm and 50 sccm, (**b**) 5 sccm and 25 sccm, and (**c**) 5 sccm and 50 sccm, respectively. Samples are placed at coordinate x = 0. Vertical lines show the end of the sample holder.

Figure 2 shows most reasonable results of the distribution of the ratio of gallium and oxygen precursors on the surface of the sample holder. This is a two-dimensional (2D) cross-section of a 3D simulation model cut parallel to the surface of the holder. The sample is placed at the coordinate x = 0, and the vertical line in each figure denotes the edge of the sample holder. The isolines show precursors ratios, where high and low values are marked by red and by blue, respectively, as also indicated on the scale on the right side of each graph. Note that the scales are different for each case.

Although the ratio of the gas flows was the same, the results of the distribution of the ratio of precursors on the sample surface show significant differences depending on the inlet gas flows. Using 10 sccm and 50 sccm for GaCl and O_2_ flows, respectively, i.e. for a gas flow ratio of 0.2, we get the precursors ratio of ~ 0.15 near the growth surface (Fig. 2a), see isolines around coordinates x = 0–0.01 and y = 0.03–0.04. With the same ratio of gas flows, but with lower inlet gas flows, i.e. 5 sccm and 25 sccm for GaCl and O_2_, respectively, the ratio of precursors on the sample growth surface is less than 0.13, as illustrated in Fig. 2b. A decrease of the gas flows ratio to 0.1 leads to a reduction of the III/VI precursor ratio below 0.07 within the growth zone (Fig. 2c). Moreover, in the latter two cases, the distribution of precursors is less uniform.

The simulation results have been used as a guide for the selection of the HVPE process parameters. Thus, we used flows of 5 sccm/25 sccm, 5 sccm/50 sccm and 10 sccm/50 sccm for HCl/O_2_ inlet gases, respectively. In addition, we tested several growth temperatures between 800 °C and 1000 °C, although the typical growth temperature for $\upbeta$-Ga_2_O_3_ is often reported to be 1050 °C^17,20^.

**Figure 3:**
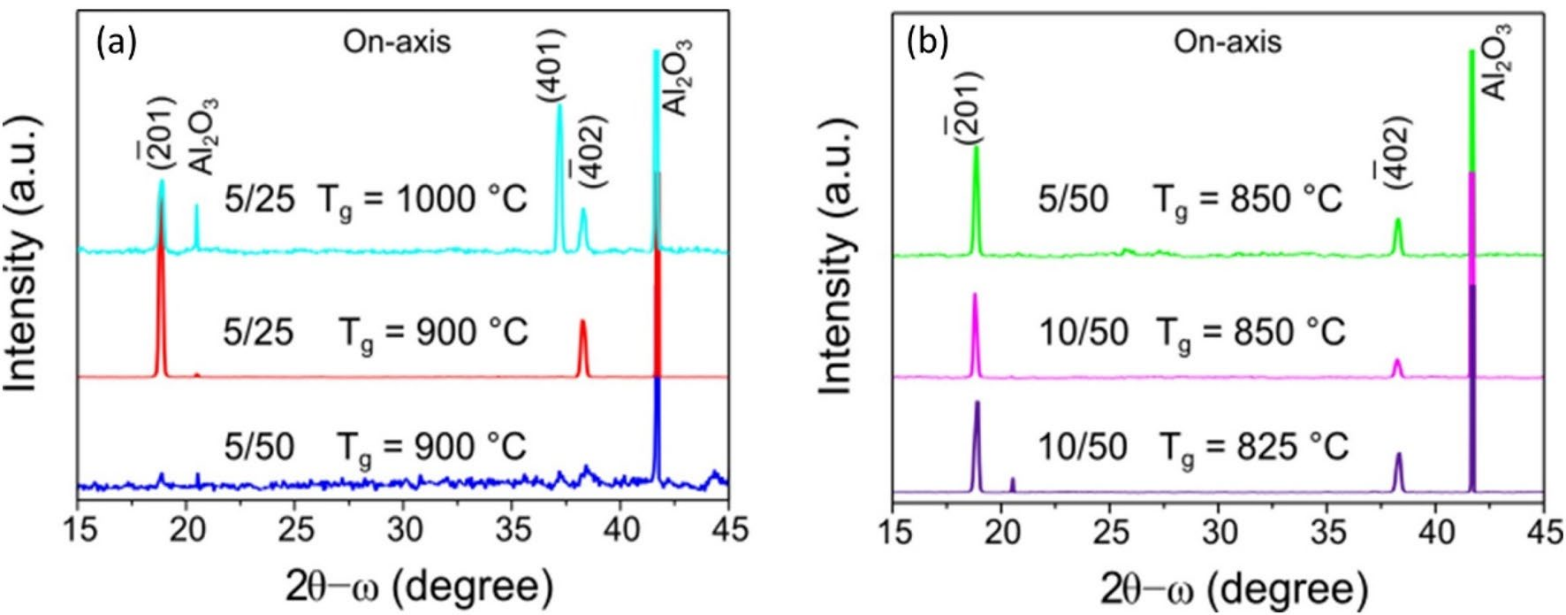
XRD spectra for Ga_2_O_3_ samples grown on sapphire (0001) with different gas flows and temperatures. Influence of (**a**) increasing oxygen flow with constant flow of HCl (**b**) increasing of HCl flow at constant oxygen flow.

We have found that the crystal quality is highly depended on the gas flow ratio, which, in turn, can be compensated by adjusting the growth temperature. On the other hand, the growth rate was almost the same as estimated from the sample thickness and it was ~ 10 µm/h. From XRD measurements we have found that good crystal quality can be achieved for growth at 850–900 °C with a ratio of 0.2 for the III/VI precursor gas flows, i.e. both 5 sccm/25 sccm and 10 sccm/50 sccm for HCl/O_2_ gas flows, respectively, can give suitable results (Fig. 3) for on-axis $\upbeta$-Ga_2_O_3_ grown on sapphire (0001). The XRD spectra have been adjusted to a sapphire reflection (0006), and for well-aligned single crystal $\upbeta$-Ga_2_O_3_ films, clear reflection peaks from the (-201) and (-402) planes appear. An increase in growth temperature or a decrease in the ratio of III/VI precursor gas flows leads to a significant degradation of the crystal quality for the Ga_2_O_3_ layers. At 1000 °C even with a precursor ratio of 0.2, the XRD spectra show an additional reflection peak from (401) plane, see example in Fig. 3a, upper spectrum. At a precursor ratio of 0.1, misorientation from the sapphire direction < 0001 > was observed for the Ga_2_O_3_ layers grown at > 900 °C, while a decrease in the growth temperature to < 850 °C could improve the structural quality, as can be seen by comparing the bottom spectrum in Fig. 3a and the upper spectrum in Fig. 3b.

**Figure 4:**
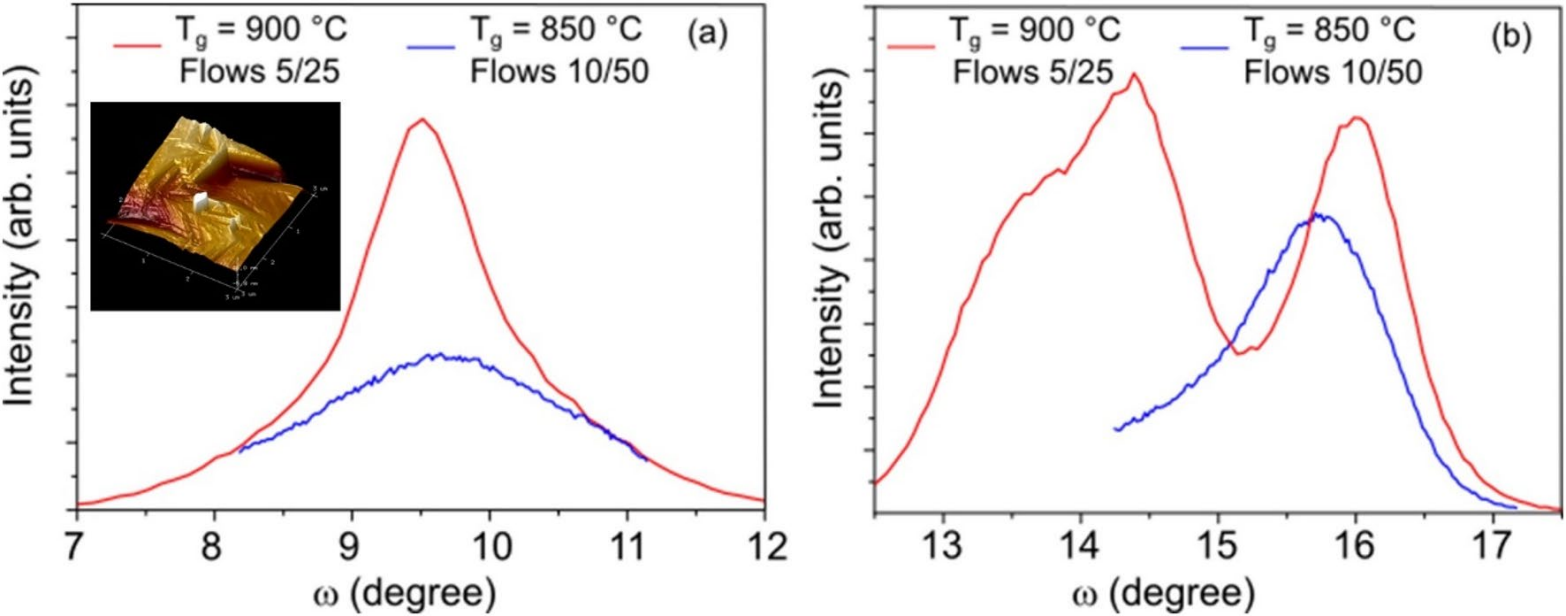
XRD omega (rocking curve) scans for the $(\overline{2}01)$ reflection peak for β-Ga_2_O_3_ films grown (**a**) on sapphire (0001) and (**b**) on off-cut sapphire substrate, respectively. Rocking curves are shown for two different process parameters as indicated for each curve. Inset in (**a**) shows 3D AFM image taken over 3 mm × 3 µm area.

For the on-axis layer produced at 900 °C with 5 sccm/25 sccm for HCl/O_2_ gas flows, the rocking curve (red line) is shown in Fig. 4a. In this case, the full width at half maximum (FWHM) was ~ 1.2 degrees, while for layers grown with different parameters or on the off-axis sapphire substrate, the FWHM is broader and can reach 2 degrees or more, as also illustrated in Fig. 4b.

In addition to the requirements for single crystallinity, flat morphology appropriate for electrical contacts was also one of the key properties for optimizing the HVPE process parameters for Ga_2_O_3_ growth. We found that a rather smooth surface can be obtained for the on-axis Ga_2_O_3_ layer grown at 825 °C with a HCl/O_2_ gas flow ratio of 10 sccm/50 sccm as illustrated by a 3D atomic force microscopy (AFM) image in the inset of Fig. 4a. The roughness was ~ 10 nm for the root mean square (RMS) for a 3 µm × 3 µm image, while the terrace height was estimated to ~ 40 nm.

**Figure 5:**
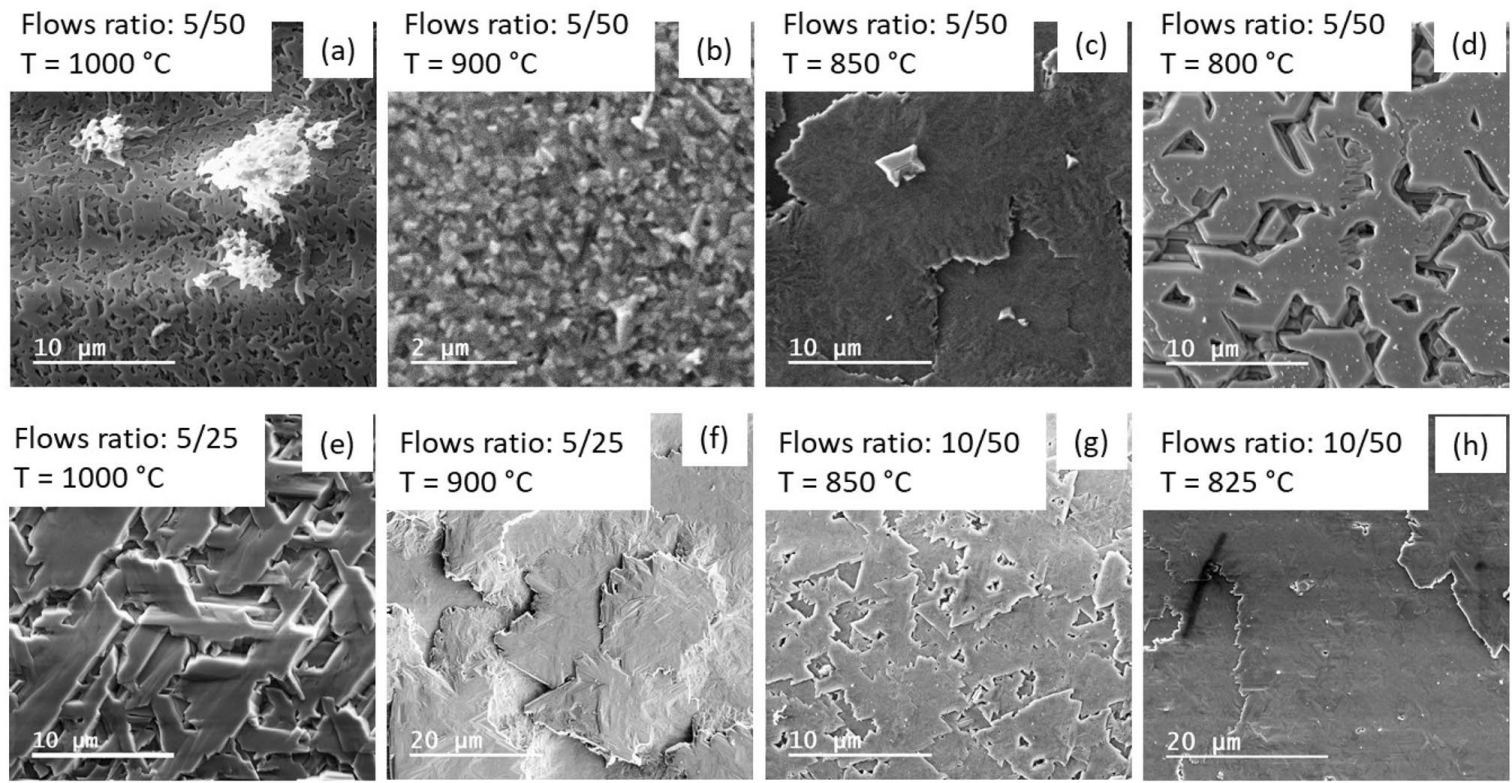
SEM images for Ga_2_O_3_ samples grown on sapphire (0 0 0 1) with different flows of precursors of Ga and oxygen: (**a**–**c**) 5 sccm and 50 sccm, respectively, (**e**,**f**) 5 sccm and 25 sccm, respectively, (**g**,**h**) 10 sccm and 50 sccm, respectively. The growth temperature was 1000 °C (**a**,**e**), 900 °C (**b**,**f**), 850 °C (**c**,**g**), 825 °C (**h**) and 800 (**d**).

Comparison of morphology quality for different samples is performed by SEM imaging and illustrated for gas flow ratios of 0.1 (Fig. 5a–d) and 0.2 (Fig. 5e–h), respectively. It is clear that even layers with good single crystallinity according to the XRD measurements could suffer from morphological degradation (for example, for samples shown in Fig. 5c–f). We have also observed that a decrease in the growth temperature results in the transformation of terraces into voids (Fig. 5c,d). The optimum between morphology and XRD results was achieved for a growth temperature of 825 °C and for a gas flow ratio of 0.2 considering our current reactor geometry. This optimized temperature is lower than typically reported for $\upbeta$-Ga_2_O_3_ growth temperature in the range of 950–1050 °C^14,17,20^.

**Figure 6:**
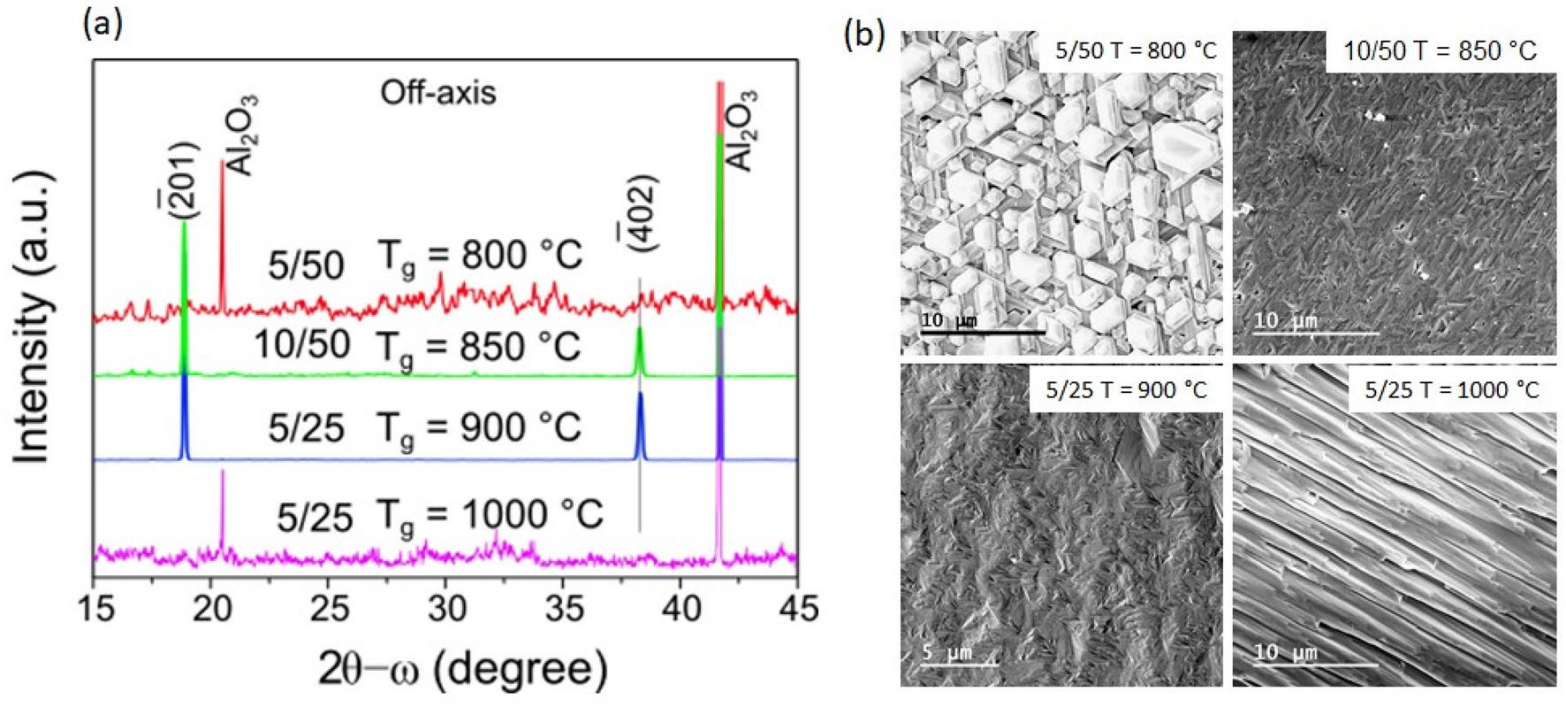
(**a**) XRD spectra are shown for several Ga_2_O_3_ samples grown with different precursors flows and at different temperatures as indicated for each spectrum. (**b**) SEM images show morphology for the same samples as in (**a**).

Morphological defects were more severe for layers grown on off-axis sapphire substrates. Even though the XRD spectra show single crystallinity when layers were grown at 850–900 °C with a gas flow ratio of 0.2 (see XRD spectra shown by blue and green in Fig. 6a), the morphology is rather rough, with RMS of ~ 200 nm as determined by AFM measurements. SEM images in Fig. 6b illustrate poor morphology for the off-axis $\upbeta$-Ga_2_O_3_ layers, which can vary from distinctly separated microcrystals for samples grown at 800 °C to stripes for a growth temperature of 1000 °C (note that the direction of the stripes is random and does not correlate with the cut-off direction of sapphire).

**Figure 7:**
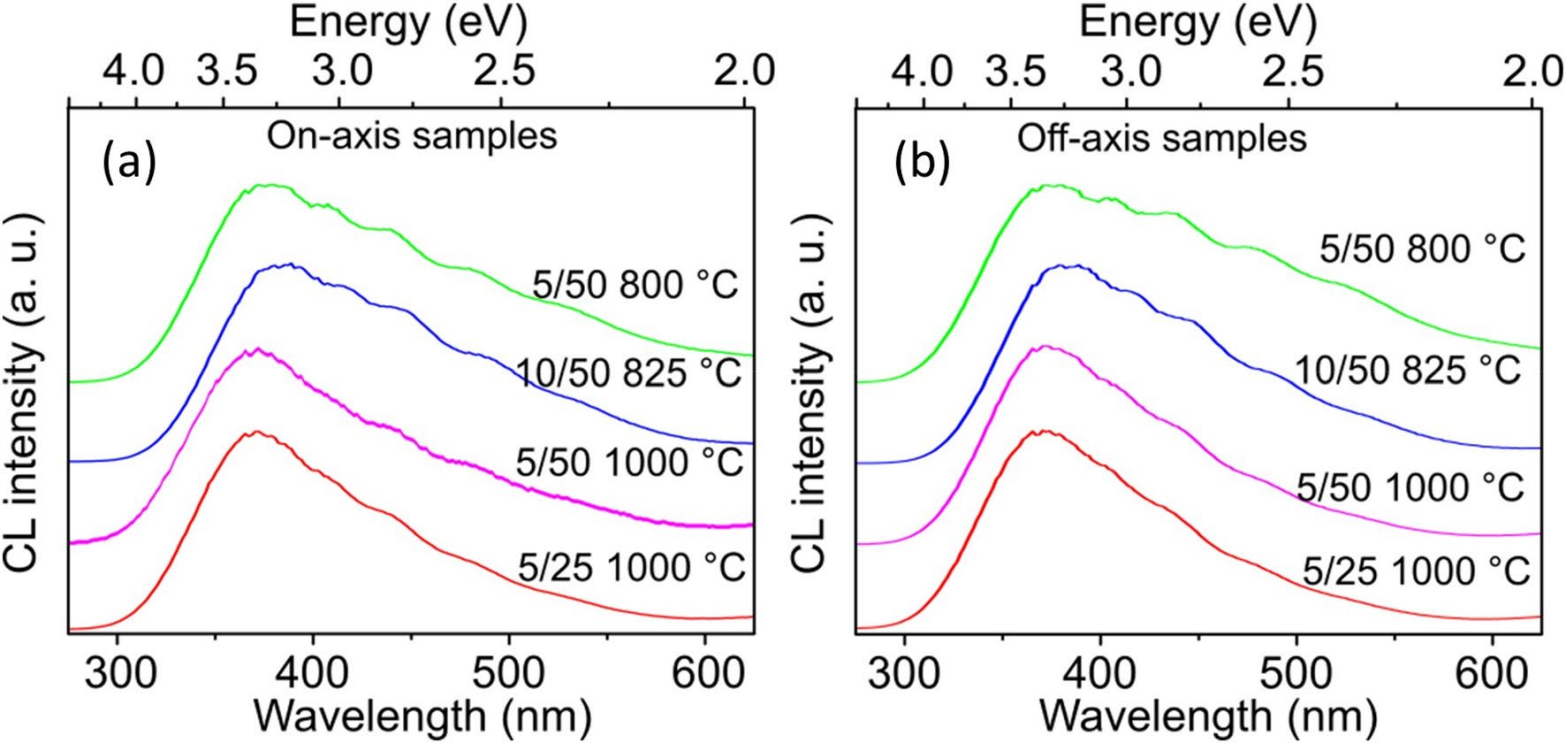
CL spectra for Ga_2_O_3_ samples grown on (**a**) sapphire (0 0 0 1) and (**b**) on 5°-off cut sapphire. Growth parameters such as gas flows of HCl/O_2_ and growth temperature are indicated for each spectrum. Spectra are normalized and shifted vertically for clarity.

Despite a significant difference in structural and morphological quality, the emission spectra were similar for all Ga_2_O_3_ layers, regardless of the substrate orientation or the parameters of the growth process. The CL spectra at room temperature shown for on- and off-axis layers in Fig. 7a,b, respectively, are dominated by a broad band with an emission peak at ~ 3.3 eV. Luminescence in Ga_2_O_3_ is not related to the band edge transition, but consists of overlapping defect-related emission lines, which appear as additional features at the lower energies compared to the 3.3 eV band. The features are more pronounced for both on- and off-axis samples grown at lower temperatures of 800–825 °C and occur at positions of ~ 3.0, ~ 2.8, ~ 2.6 eV, and 2.4 eV respectively.

The studied HVPE Ga_2_O_3_ samples were unintentionally *n*-type doped; thus, both intrinsic defects and impurities can be responsible for the origin of the defect luminescence. There have been numerous studies related to the emission in $\upbeta$-Ga_2_O_3_. The band at ~ 3.3 eV is so-called UV emission, which has been discussed previously and assigned to transitions between electrons and self-trapped holes^4,6^. The blue bands with peaks at ~ 2.8 and ~ 3.0 eV, respectively, are attributed to the transitions between deep donors and acceptors, the role of which is played by O and Ga vacancies, respectively^21^. The feature in our CL spectra at ~ 2.4 eV is close to the green emission at 2.5 eV assigned to excitons bound to intrinsic defects or impurities^22^. We have not observed any dominant red emission at ~ 2.0 eV associated with nitrogen doping^23,24^, since N_2_ was used only as a carrier gas during the growth and did not incorporated in the Ga_2_O_3_ layers. Recently, Ho et al. used a model with an optimized Koopmans-compliant hybrid functional allowing to explain different emissions in $\upbeta$-Ga_2_O_3_ by the recombination of the bound exciton, where a hole is trapped by intrinsic acceptor levels while electron is weakly localized^25^. Accordingly, calculations of the transition involving a hole trapped by a divacancy (${V}_{Ga}+{V}_{O}$) with a tetrahedrally-coordinated gallium site and threefold-coordinated oxygen site resulted in the energy of 2.9 eV, which corresponds to the blue emission at ~ 3.0 eV in the experimental spectra. The energy of the recombination involving a gallium vacancy with octahedrally-coordinated sites (${V}_{Ga}^{2-}$) was calculated as 2.7 eV, which correlates well with another blue emission line at ~ 2.8 eV observed in the experiment. The transition energy of 2.3 eV was obtained for the hole trapped on interstitial oxygen (neutral ${O}_{i}^{0}$) and corresponds to the green emission at ~ 2.4 eV, which especially clearly observed in the $\upbeta$-Ga_2_O_3_ samples treated under oxygen-rich conditions^22^. This assignment of the green line is in a good agreement with our results. As can be seen in Fig. 7, the green emission is more pronounced for the samples grown at oxygen-rich conditions, i.e. at lower temperatures and especially with a higher oxygen gas flow (i.e. with a Ga/O gas flow ratio of 5/50). The feature at ~ 2.6 eV is still to be explain and, apparently, further studies are needed to clarify the origin of different emission lines in the visible region in $\upbeta$-Ga_2_O_3_ samples.

**Figure 8:**
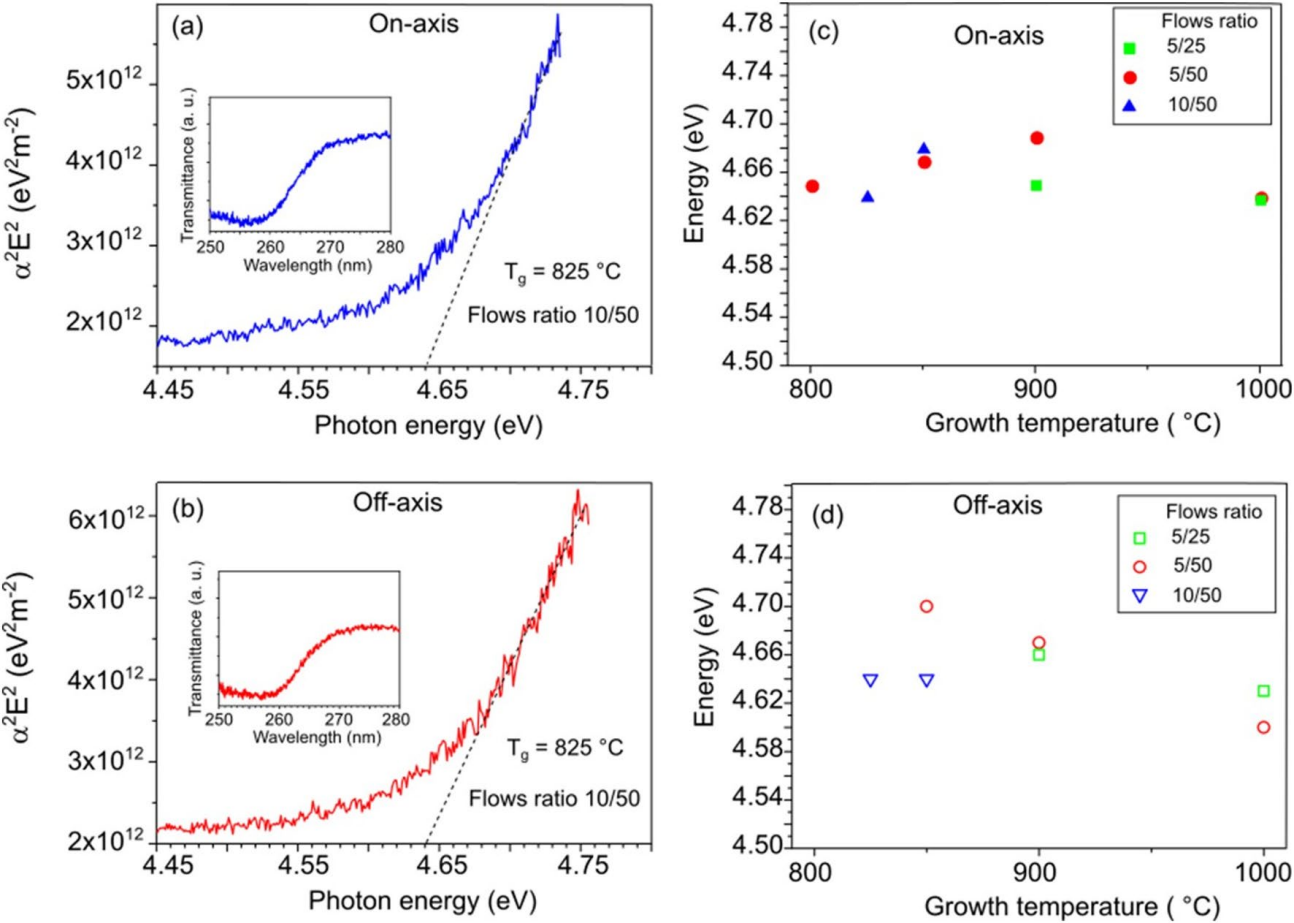
Examples of the squared absorption coefficient plotted as a function of photon energy for Ga_2_O_3_ layers grown on (**a**) on-axis sapphire and (**b**) off-axis sapphire. Corresponding transmission spectra are shown in inset. The estimated band gap energy for (**a**) on- and (**b**) off-axis Ga_2_O_3_ samples grown at different temperature and with different precursor gas flow ratio.

We have estimated the optical band gap in the $\upbeta$-Ga_2_O_3_ layers from transmission measurements. The absorption coefficient $\alpha$ for the direct band gap semiconductors is related to the band gap energy *E*_*g*_ as $\alpha E\sim \sqrt{E-{E}_{g}}$, where *E* is the photon energy. Examples of the dependence of $(\alpha {E)}^{2}$ on the photon energy for on- and off-axis samples grown at 825 °C with a Ga/O_2_ gas flow ratio of 10/50 are shown in Fig. 8a,b. The corresponding transmission spectra are shown in the insets. Although a sufficiently steep edge is not observed in the absorption spectra due to defects and impurity states contributing to the tail below the band gap, we can still estimate *E*_*g*_ by extrapolating the linear part of $(\alpha {E)}^{2}$ to zero. The extracted values of *E*_*g*_ are plotted in Fig. 8c,d for on- and off-axis $\upbeta$-Ga_2_O_3_ layers fabricated under different process conditions. As can be seen, there is only a small difference within the random error for the *E*_*g*_ estimates with an average value of ~ 4.65 eV. This value is less than 4.8–4.9 eV recently obtained for the band gap energy in heteroepitaxial $\upbeta$-Ga_2_O_3_^13,14^, but close to 4.68 eV obtained for homoepitaxial $\upbeta$-Ga_2_O_3_ grown at 700 °C^11^. The reduced band gap energy reflects likely the effect of high defect density such as vacancies and vacancy clusters in HVPE $\upbeta$-Ga_2_O_3_ layers^26^.

## Conclusion

We optimized the HVPE process to produce $\upbeta$-Ga_2_O_3_ layers by simulating the ratio of gallium to oxygen precursors inside the growth chamber and compared the results for (0001) and 5 degrees off-axis sapphire substrates. We found that the structural quality, as measured by XRD, was good for both on- and off-axis samples grown at 850–900 °C and with a III/VI precursor ratio of 0.2; however, the morphology of off-axis $\upbeta$-Ga_2_O_3_ layers was significantly worse. A decrease in the ratio of precursors or an increase in growth temperature to 1000 °C degrades the crystalline quality of layers with both orientations. In addition to the precursor ratio, the morphology of the layers was also influenced by inlet gas flows. So far, the best results in terms of single crystallinity and morphology have been obtained when grown at 825 °C and with inlet gas flows of 10 and 50 sccm for HCl and O_2_, respectively (i.e. precursor ratio 0.2). The optical characterization has shown little difference between samples of different qualities and orientations, with a typical emission maximum at ~ 3.3 eV corresponding to the recombination of electrons with self-trapped holes in unintentionally doped *n*-type $\upbeta$-Ga_2_O_3_. The main difference was observed in an increase in blue and green emissions when the layers were grown at lower temperatures of 800–825 °C. The absorption edge was broadened due to the likely influence of a high density of intrinsic defects, such as vacancies, which led to a decrease in energy of the band gap to ~ 4.65 eV.

## Methods

### Growth modelling

To optimize the growth parameters such as precursor ratio, we have used the transport model based on numerical solution of the nonlinear coupled partial differential equations for the conservation of mass (i.e. continuity equation), energy, momentum (i.e. Navier–Stokes equation), and individual species. For calculations, we have used COMSOL Multiphysics software with CAD Import Module, CFD Module and Chemical Reaction Engineering Module.

The void in the model (Fig. 1b) means empty space without gas flows behind the sample holder since the sample holder has an inclination angle. This was done to speed up the calculations. Also, to reduce the time for simulations, the model has been treated as a three-dimensional problem with a vertical mirror plane drawn through the center of the reactor. The following equations have been used to describe the system:

1 $$\nabla \cdot {\mathbf{j}}_{i} + \rho \left( {{\mathbf{u}} \cdot \nabla } \right)\omega_{i} = R_{i}$$

where *ρ* is a density of gas, **u**—is velocity and *ω* and *R* are cylindrical coordinates, respectively.

Species continuity equation is written as:

2 $${\mathbf{N}}_{i} = {\mathbf{j}}_{i} + \rho {\mathbf{u}}\omega_{i}$$

where

3 $${\mathbf{j}}_{i} = - \left( {\rho \omega_{i} \mathop \sum \limits_{k} D_{ik} {\mathbf{d}}_{k} + D_{i}^{T} \frac{\nabla T}{T} } \right)$$

We denote:

4 $${\mathbf{d}}_{k} = \nabla \chi_{k} + \frac{1}{{p_{A} }}\left[ {\left( {\chi_{k} - \omega_{k} } \right)\nabla p_{A} + \frac{{\rho \omega_{k} z_{k} }}{{M_{k} }}F\nabla V - \mathop \sum \limits_{l} \frac{{\rho \omega_{l} z_{l} }}{{M_{l} }}F\nabla V} \right],$$

and.

5 $$\chi_{k} = \frac{{\omega_{k} }}{{M_{k} }}M_{n } , \quad M_{n } = \left( {\mathop \sum \limits_{i} \frac{{\omega_{i} }}{{M_{i} }}} \right)^{ - 1}$$

Navier–Stokes (momentum) equation reads as:

6 $$\rho \left( {{\mathbf{u}} \cdot \nabla } \right){\mathbf{u}} = \nabla \cdot \left[ { - \rho {\mathbf{I}} + \mu \left( {\nabla {\mathbf{u}} + \left( {\nabla {\mathbf{u}}} \right)^{T} } \right) - \frac{2}{3}\mu \left( {\nabla \cdot {\mathbf{u}}} \right){\mathbf{I}}} \right] + {\mathbf{F}}$$

and mass equation:

7 $$\nabla \cdot \left( {\rho {\mathbf{u}}} \right) = 0$$

The flow velocities have been obtained from the energy Eq. (1) and momentum Eq. (6), while the gas concentration profiles are obtained from the conservation of mass, Eq. (7) and individual species Eq. (2). In the calculations, the flow was assumed to be incompressible.

Transport properties of the gases are calculated from the kinetic theory of gases. The species included in the calculations are GaCl, O_2_, and N_2_. The dynamic viscosity and diffusion coefficients are calculated from the kinetic theory of gases using Lennard–Jones parameters (see Table 1) taken from literature^27,28^.

**Table 1:** The following Lennard–Jones parameters was used in the simulation model for calculation of dynamic viscosity and diffusion coefficients.

Species	Mass (Kg/mol)	σ (Å)	ε/k (K)	References
GaCl	0.10518	5.47	378.2	^19^
N_2_	0.028	3.798	71.4	^20^
O_2_	0.032	3.467	106.6	^20^

The differential equations were solved using mesh shown in Fig. 1b. The mesh was denser in the places of interest, i.e. near the gas pipes and sample holder and less dense after the sample holder and closer to the quartz tube edges.

### Growth

We used a horizontal HVPE reactor containing a quartz tube with two boats for metal precursors and the furnace with three temperature zones. The growth zone consists of a rectangular tube with an inclined sample holder. Applied resistive heating allows to increase temperature up to 1050 °C. To control the growth temperature and gallium boat temperature, we used two thermocouples inserted inside the chamber close to the sample holder and the boat, respectively. We used two orientation of sapphire substrates: (0001) and 5-degree off-axis cut in direction to *m*-plane. Samples have been grown with different precursor flows at normal atmospheric pressure and different temperatures between 800 °C and 1000 °C. GaCl and O_2_ gases have been used as III and VI precursors, respectively. GaCl was obtained in-situ by flowing HCl gas through the boat with liquid metallic Ga kept at 850 °C. The flow of the carrier gas, N_2_, was always kept to 1500 sccm/min. In our present reactor configuration, the HCl flow could be varied within only a small range between 5 and 10 sccm.

### Characteriztion

The crystal structure was investigated by XRD (PANalytical X’Pert Pro) using Cu-Kα radiation. AFM measurements were done using instrument Dimension 3100 SPM with the VT-102 vibration isolation table. For transmission (absorption) measurements, we have used deuterium lamp as a light source. Transmission spectrum from the pure sapphire substrate served as an instrumental function. Samples surface morphology was studied using Leo 1500 Gemini SEM linked to the MonoCL4 system for investigation of luminescence spectrum. CL measurements were done at room temperature with the electron beam acceleration voltage of 5 kV.
